# Identification of Potential Biomarkers and Immune Cell Signatures in COVID-19 Myocarditis Through Bioinformatic Analysis

**DOI:** 10.1155/crp/2349610

**Published:** 2025-04-07

**Authors:** Yongfei Song, Xiaofei Wang, Dongdong Tong, Xiaoyan Huang, Xiaojun Jin, Chuanjing Zhang, Jianhui Liu, Bo Guo, Chen Huang, Jiangfang Lian

**Affiliations:** ^1^Ningbo Institute of Innovation for Combined Medicine and Engineering, Ningbo Medical Center Lihuili Hospital, Ningbo University, Ningbo, Zhejiang, China; ^2^Department of Cardiology, Ningbo Medical Center Lihuili Hospital, Ningbo University, Ningbo, Zhejiang, China; ^3^Biomedical Experimental Center, Xi'an Jiaotong University, Xian, Shaanxi, China; ^4^Department of Cell Biology and Genetics, School of Basic Medical Sciences, Xi'an Jiaotong University Health Science Center, Xian, Shaanxi, China; ^5^Key Laboratory of Environmentally and Genetically Associated Diseases, Xi'an Jiaotong University, Ministry of Education, Xian, Shaanxi, China

**Keywords:** bioinformatics, biomarker, COVID-19 myocarditis, immune infiltration, viral myocarditis

## Abstract

**Objective:** The present study aims to elucidate the significance of immune cell infiltration in Coronavirus disease 2019 (COVID-19) myocarditis and identify potential diagnostic markers for this condition. Myocarditis, an inflammatory cardiac disease, primarily results from viral infections. Although the association between COVID-19 and myocarditis is well-established, the specific mechanism(s) underlying this relationship remain incompletely understood.

**Methods:** The GSE53607 and GSE35182 datasets were obtained from the GEO database, which contains samples from a mouse model for viral myocarditis. Differentially expressed genes (DEGs) and candidate biomarkers were selected using the LASSO regression model and support vector machine recursive feature elimination (SVM-RFE) analysis. Subsequently, the diagnostic potential of these biomarkers was evaluated by calculating the area under the receiver operating characteristic curve (AUC). Further validation of the biomarkers was conducted using the GSE183850 dataset, which consists of samples from patients with COVID-19 myocarditis. In addition, CIBERSORT analysis was employed to estimate the compositional patterns of 22 types of immune cell fractions in merged cohorts.

**Results:** Thirty genes were identified, with a significant proportion of the DEGs being associated with carbohydrate binding, endopeptidase activity, and pathogenic organisms such as *Staphylococcus aureus* and coronavirus disease. Importantly, gene sets related to the IL6-JAK-STAT3 signaling pathways, inflammatory response, and interferon response exhibited differential activation in viral myocarditis compared to the control group. In addition, in the context of COVID-19 myocarditis patients from the GSE183850 dataset, B2M and C3 were established as diagnostic markers that were subsequently validated (AUC = 0.978 and AUC = 0.956, respectively). Furthermore, analysis of immune cell infiltration revealed correlations between B2M and C3 expression levels and the activation of NK cells, dendritic cells, T cells CD4 memory resting, as well as eosinophils.

**Conclusion:** B2M and C3 have been identified as potential biomarkers for viral myocarditis, providing valuable insights for future investigations into the pathogenesis of COVID-19-associated myocarditis.

## 1. Introduction

Myocarditis, an inflammatory cardiac disease, is primarily caused by viral infections and affects individuals of all age groups, ethnicities, and genders. According to the latest Global Burden of Disease statistics, the global prevalence of myocarditis is estimated to range from 10.2 to 105.6 cases per 100,000 individuals [1, 2]. Epidemiological studies have highlighted viral infections as a leading cause of myocarditis, with SARS-CoV-2 infection indicating a ≥ 15-fold increased risk compared to other causes [3]. In the United States, the incidence rate of myocarditis because of SARS-CoV-2 infection has been estimated at approximately 150 cases per 100,000 individuals compared to 9 cases per 100,000 in non-COVID cases during the same period reported by the Centers for Disease Control and Prevention [4]. It should be noted that currently there are no clinically available specific blood tests for confirming myocarditis diagnosis [5], and endomyocardial biopsy (EMB), which serves as the gold standard for definitive diagnosis, was typically not performed during the pandemic because of concerns about viral transmission [6, 7]. Consequently, the diagnosis of COVID-19-associated myocarditis has relied more heavily on clinical symptoms and elevated troponins levels. This diagnostic approach is further complicated by similarities between COVID-19-associated myocarditis and other forms of viral myocarditis and pericarditis. Presenting symptoms include fever, cough, chest pain/pressure, dyspnea, palpitations, and syncope, making it even more challenging to diagnose COVID-19-related myocarditis [8–10].

In recent years, the integration of gene expression microarray analysis and bioinformatics has been extensively utilized to identify potential diagnostic biomarkers and comprehend their roles in cardiovascular disease (CVD) [11]. This approach aims to further investigate the pathogenesis and develop potential therapies [12, 13]. Mounting evidence suggests that immune cell infiltration plays a pivotal role in the occurrence of myocarditis [14]. Specifically, patients with myocarditis exhibit significant immune modulation, characterized by alterations in B and T lymphocytes, macrophages, and eosinophils cells [15]. In addition, there is an observed elevation in circulating levels of several chemokines and cytokines [16]. Despite this wealth of evidence, only a limited number of studies have employed CIBERSORT to explore immune cell infiltration in myocarditis, particularly within the context of coronavirus-induced myocarditis. Furthermore, few investigations have aimed to identify potential diagnostic markers for patients with COVID-19-associated myocarditis. Therefore, this represents a crucial avenue for further research in this field.

This study involved acquiring two microarray datasets of a mouse model for viral myocarditis from the GEO database. The datasets were merged to create a meta-data cohort that facilitated identifying differentially expressed genes (DEGs) between viral myocarditis samples and controls. Machine-learning algorithms were then applied to filter and determine diagnostic biomarkers for viral myocarditis. In addition, candidate genes strongly associated with immune infiltration underwent validation in a human high-throughput sequencing validation cohort before being used to develop a diagnostic prediction model for SARS-CoV-2 infected samples through logistic regression analysis. Furthermore, the investigation explored immune infiltration in COVID-19 myocarditis using the CIBERSORT method, offering potential insights for future research.

## 2. Materials and Methods

### 2.1. Microarray Data

The GSE53607 [17] and GSE35182 datasets [18] were obtained from the NCBI Gene Expression Omnibus (GEO) database (https://www.ncbi.nlm.nih.gov/geo/). Both datasets were based on the GPL6246 platform of Affymetrix Mouse Gene 1.0 ST Array. The GSE53607 dataset involved the collection of hearts from five mice intraperitoneally infected with TMEV on days 4, 7, and 60 post-infection for RNA isolation and hybridization on Affymetrix microarrays. Meanwhile, the GSE35182 dataset consisted of a total of 12 samples, including infected males, infected females, uninfected males, and uninfected females with three mice in each group. To facilitate further integration analysis, these two datasets were merged into a metadata cohort because of their shared platform and significance in combining data from different sources. Furthermore, batch effect was eliminated using the combat function of the “SVA” package in R software [19]. In addition, for validation purpose in RNA sequencing on the GPL24676 platform of Illumina NovaSeq 6000, we utilized the GSE183850 dataset [20], which comprised donated cardiac pericytes inoculated with SARS-CoV-2 mNeonGreen at a multiplicity of infection (MOI) of 1 (five technical replicates/donor) or mock samples (three technical replicates/donor).

### 2.2. Identification of DEGs

The combat function of the SVA package was initially applied to preprocess and minimize any potential biases caused by batch effects on the merged metadata cohort, which consisted of two datasets. Subsequently, we utilized the limma package [21] from R (https://www.bioconductor.org/) for background correction, array normalization, and conducting differential expression analysis. Specifically, samples meeting the threshold criteria of an adjusted *p* value < 0.05 and |log fold change (FC)| > 1.2 were employed for identifying DEGs.

### 2.3. Functional Enrichment Analysis

The DEGs were annotated and analyzed for their biological functions using Gene Ontology (GO) analysis, Kyoto Encyclopedia of Genes and Genomes (KEGG) pathway analysis, and Gene Set Enrichment Analysis (GSEA). GO analysis provides a hierarchical controlled vocabulary divided into three subsets: cellular component, biological process, and molecular function. Similarly, KEGG pathway analysis includes pathway maps illustrating molecular interactions, reactions, and networks. GSEA is a computational approach that assesses the statistical significance of differences between two groups based on a predefined gene set. For conducting GO and KEGG analyses, we utilized R software along with the “clusterProfiler” package [22], while GSEA analysis was performed using the GSEA software (R package version 1.60.0) [23]. The reference gene set employed was “c2.cp.kegg.v7.0.symbols.gmt” from the Molecular Signatures Database (MSigDB). An enrichment was considered significant if the *p* value was less than 0.05 with a false discovery rate below 0.025.

### 2.4. Candidate Diagnostic Biomarker Screening

The prediction disease status and identification of significant prognostic variables were conducted using two machine learning algorithms. We employed the least absolute shrinkage and selection operator (LASSO) as the first algorithm, which is a regression analysis technique that utilizes regularization to enhance prediction accuracy. This algorithm was implemented using the “glmnet” package [24] in R programming language. As for the second algorithm, we utilized support vector machine (SVM), a widely adopted supervised machine-learning method for classification and regression tasks. To mitigate overfitting, we applied the recursive feature elimination (RFE) algorithm to select the optimal genes from the meta-data cohort [25]. Consequently, we deployed SVM-RFE to identify genes with superior discriminative power. Finally, we integrated overlapping genes identified by these two algorithms and validated their expression levels in the GSE183850 dataset.

### 2.5. Diagnostic Value of Feature Biomarkers in COVID-19 Myocarditis

The mRNA expression data were utilized to construct the ROC curve for evaluating the predictive capacity of the identified biomarkers. Subsequently, the AUC value derived from the ROC curve was employed as a measure of diagnostic effectiveness and validated using the GSE183850 dataset.

### 2.6. Discovery of Immune Cell Subtypes

The bioinformatics algorithm CIBERSORT, available at https://cibersortx.stanford.edu/, was employed to quantify the infiltration of immune cells based on gene expression profiles in the analysis of COVID-19 myocarditis samples. This algorithm estimated the putative abundance of distinct subtypes of immune cells using a reference set comprising 22 types. Furthermore, correlation analysis and visualization were conducted for these 22 infiltrating immune cells types using the R package “corrplot” [26], while variations in immune cell infiltration between the COVID-19 myocarditis and control samples were illustrated through violin plots generated by the “vioplot” package [27] in R.

### 2.7. Correlation Analysis Between Identified Genes and Immune Cells

The relationship between immune cells and feature genes was investigated using Spearman's rank correlation analysis in R software. Visualization of the plot was achieved employing the “ggplot2” package [28]. Statistical significance was defined as a *p* value < 0.05.

### 2.8. Statistical Analysis

Statistical analysis was conducted using R (version 3.6.3). Group comparisons for continuous variables were performed with Student's *t*-test for normally distributed variables and the Mann–Whitney *U*-test for nonnormally distributed variables. LASSO regression analysis was carried out using the “glmnet” package [24], while the SVM algorithm was implemented with the e1071 package [29] in R. Diagnostic efficacy of the included biomarkers was assessed through ROC curve analysis, and Spearman's correlation was employed to analyze gene biomarkers expression and infiltrating immune cells' relationship. It is important to note that a statistically significant threshold of adjusted *p* value < 0.05 determined the result significance.

## 3. Results

### 3.1. Identification of DEGs in Mouse Model for Viral Myocarditis

**Figure 1:**
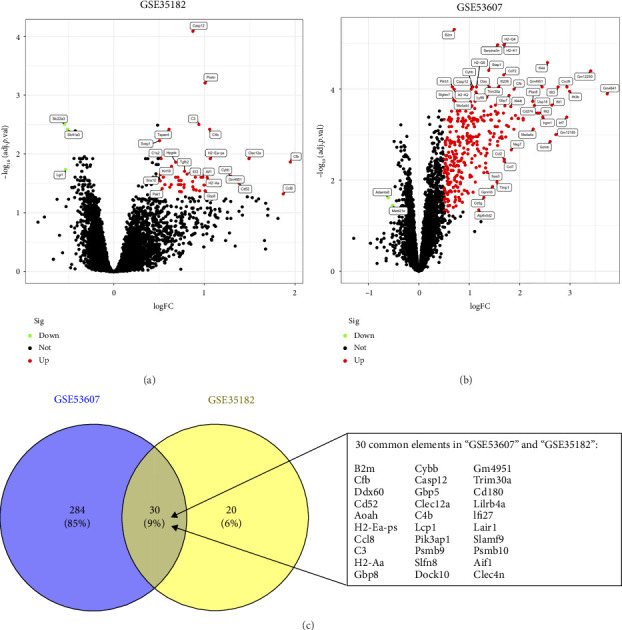
The volcano map of DEGs between myocarditis samples and control samples in mouse model obtained from (a) GSE35182 and (b) GSE53607 datasets. The green dots represent downregulated DEGs; the red dots represent the upregulated DEGs; black dots indicate the remaining genes that were not significantly changed. (c) Venn diagram demonstrating 30 DEGs shared in two datasets.

In this study, the expression values from two GEO datasets (GSE53607 and GSE35182) were normalized and subsequently visualized as volcano plots (Figures 1(a) and 1(b)). After removing batch effects, DEGs in the metadata were analyzed using the limma package. The Venn Diagram (Figure 1(c)) demonstrated that there were 30 genes with highly significant logFC present in both datasets, thus prompting further investigation to identify potential biomarkers.

### 3.2. Functional Enrichment Analysis of DEGs

**Figure 2:**
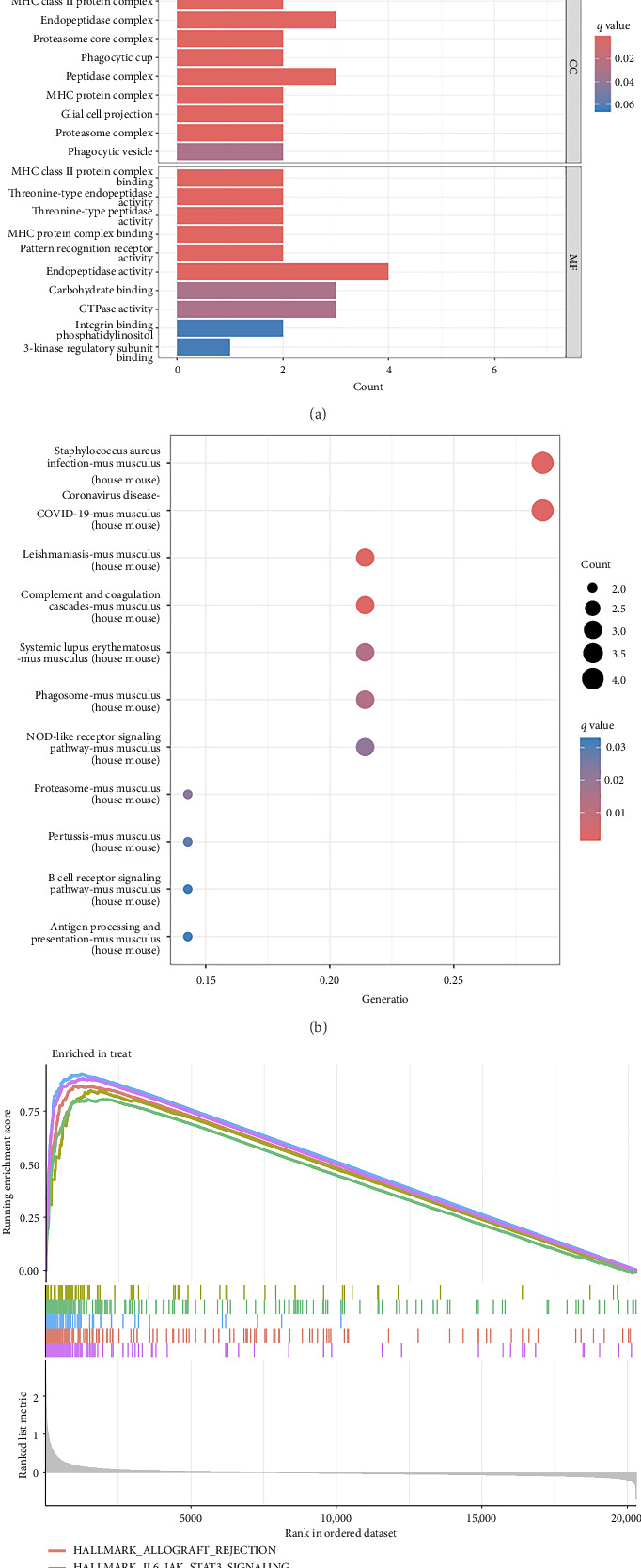
The functional enrichment analysis to identify potential biological processes of differentially expressed genes. (a) The GO enrichment analysis. (b) The KEGG enrichment analysis. (c) The gene set enrichment analysis.

The functional enrichment analyses revealed that the DEGs were predominantly associated with the regulation of immune effector processes, lymphocyte proliferation, MHC class II protein complex binding, carbohydrate binding, integrin binding, and endopeptidase activity, as depicted in Figure 2(a). Moreover, the KEGG enrichment analysis indicated significant involvement in *Staphylococcus aureus* infection, complement and coagulation cascades, systemic lupus erythematosus, antigen processing and presentation, B cell receptor signaling pathway, and coronavirus disease, as demonstrated in Figure 2(b). In addition, GSEA results revealed that the enriched pathways primarily correlated with allograft rejection, activation of IL6-JAK-STAT3 signaling pathways, inflammatory response, and interferon response, as illustrated in Figure 2(c). Overall, the findings from functional enrichment analyses underscore the pivotal role of immune response in viral myocarditis.

### 3.3. Selection and Validation of Diagnostic Feature Genes

**Figure 3:**
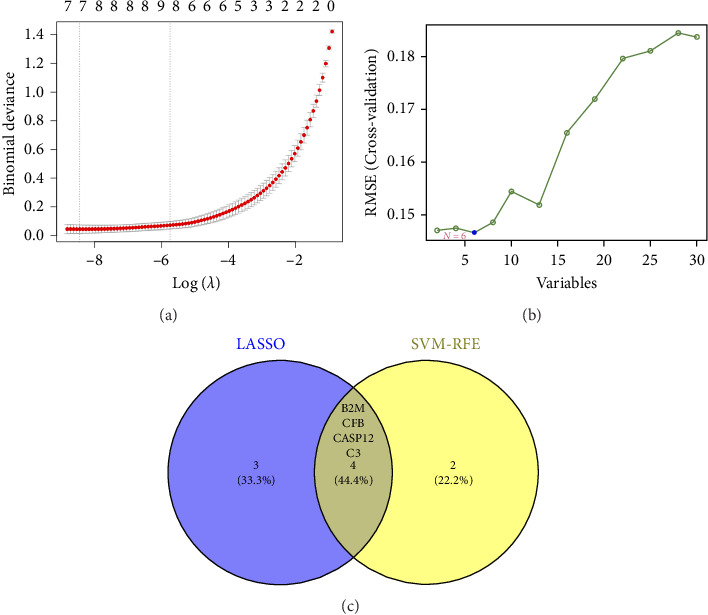
Screening process of diagnostic biomarker candidates for myocarditis. (a) Tuning feature selection in the least absolute shrinkage and selection operator model. (b) A plot of biomarkers selection via support vector machine-recursive feature elimination (SVM-RFE) algorithm. (c) Venn diagram demonstrating four diagnostic markers shared by the least absolute shrinkage and selection operator and SVM-RFE algorithms.

**Figure 4:**
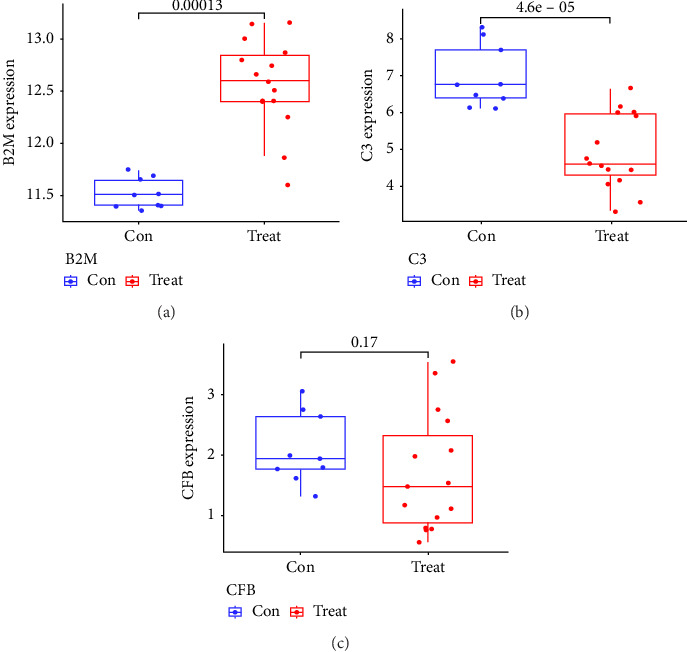
Validation of the expression of diagnostic biomarkers in the GSE183850 dataset. (a) B2M. (b) C3. (c) CFB.

Two distinct algorithms were employed to screen potential biomarkers for viral myocarditis. Firstly, the LASSO regression algorithm was utilized to narrow down the DEGs, resulting in the identification of seven variables as diagnostic biomarkers (Figure 3(a)). Subsequently, the SVM-RFE algorithm was employed to determine a subset of six features among the DEGs (Figure 3(b)). Ultimately, four overlapping features, namely, B2M, C3, CFB, and CASP12, were selected as potential biomarkers for viral myocarditis using both algorithms (Figure 3(c)). Furthermore, to ensure the accuracy and reliability of our findings, we validated the expression levels of the four features using COVID-19 myocarditis patient samples from dataset GSE183850. It is noteworthy that Casp12 could not find a matched gene in the human genome because of species differences. Amongst the remaining three genes, B2M exhibited significantly higher expression levels in SARS-CoV-2 mNeonGreen inoculated samples compared to control samples (Figure 4(a), *p* < 0.001). Conversely, C3 showed significantly lower expression levels in SARS-CoV-2 mNeonGreen inoculated samples compared to control samples (Figure 4(b), *p* < 0.001). However, no significant difference was observed between groups regarding CFB expression (Figure 4(c)). Consequently, the results suggest that these three genes may hold potential value in developing a diagnostic model using logistic regression algorithm within this metadata cohort.

### 3.4. Diagnostic Effectiveness of Feature Biomarkers in COVID-19 Myocarditis

**Figure 5:**
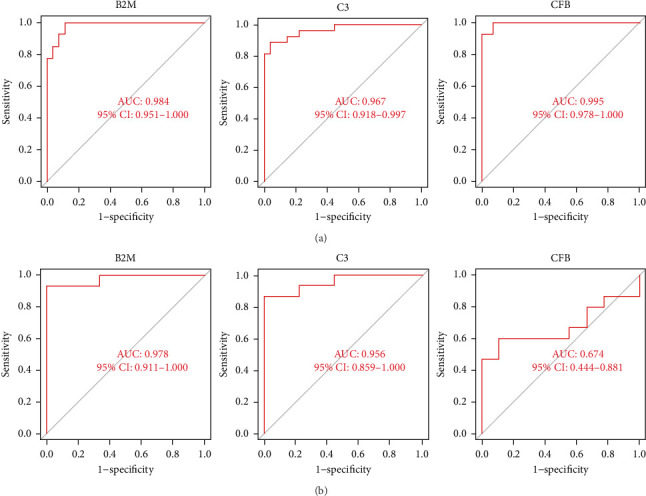
The receiver operating characteristic (ROC) curve of the diagnostic effectiveness of the three diagnostic markers. (a) ROC curve of B2M, C3, and CFB in the metadata cohort. (b) ROC curve of B2M, C3, and CFB in the GSE183850 dataset.

The three biomarkers demonstrated promising diagnostic capabilities in distinguishing myocarditis from control samples. B2M exhibited an AUC of 0.984 (95% CI 0.951–1.000), C3 presented an AUC of 0.967 (95% CI 0.918–0.997), and CFB showed an AUC of 0.995 (95% CI 0.978–1.000), as depicted in Figure 5(a), highlighting their favorable diagnostic value for myocarditis detection. Moreover, the robust discriminatory ability of these biomarkers was confirmed by the GSE183850 dataset, with B2M exhibiting an AUC of 0.978 (95% CI 0.911–1.000), C3 presenting an AUC of 0.956 (95% CI 0.859–1.000), and CFB showing a lower performance with only an AUC of 0.674 (95% CI 0.444–0.881), as shown in Figure 5(b). These findings collectively indicate that these biomarkers are highly effective for diagnostic applications.

### 3.5. Immune Infiltration Analysis

**Figure 6:**
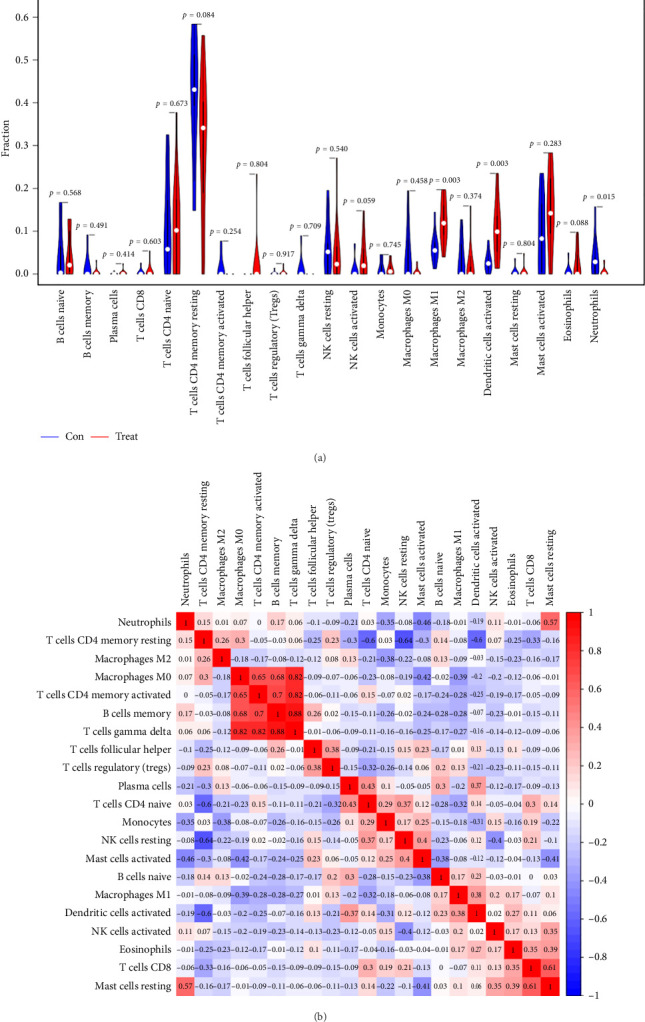
Distribution and visualization of immune cell infiltration. (a) Comparison of 22 immune cell subtypes between COVID-19 myocarditis samples and control samples. Blue and red colors represent control and COVID-19 myocarditis samples, respectively. (b) Correlation matrix of all 22 immune cell subtype compositions. Both horizontal and vertical axes demonstrate immune cell subtypes. Immune cell subtype compositions (higher, lower, and same correlation levels are displayed in red, blue, and white, respectively).

The CIBERSORT algorithm was employed to predict the infiltration of immune cell between SARS-CoV-2 mNeonGreen inoculated and mock control samples, following the observation of a pronounced enrichment in immune response during the functional enrichment analysis of DEGs. The resulting vioplot revealed notable disparities in the proportions of specific immune cells. Remarkable, compared to the control samples, the SARS-CoV-2 mNeonGreen samples exhibited significantly elevated proportions of activated NK cells (*p*=0.050), macrophage M1 cells (*p*=0.003), and activated dendritic cells (*p*=0.003). Conversely, there was a significant decrease in neutrophils proportion in the SARS-CoV-2 mNeonGreen samples compared to the control samples (*p*=0.015) (Figure 6(a)). The correlation between 22 types of immune cells was calculated and presented in Figure 6(b). The correlation heatmap revealed several significant association among immune cells. Specifically, memory B cells exhibited strong positive correlations with memory CD4 T cells (*r* = 0.7, *p* < 0.001), gamma delta T cells (*r* = 0.88, *p* < 0.001), and macrophages M0 cells (*r* = 0.68, *p* < 0.001). Similarly, Plasma cells showed a significant positive correlation with naive CD4 T cells (*r* = 0.43, *p*=0.034). In addition, CD8 T cells displayed a significant positive correlation with resting mast cells (*r* = 0.61, *p*=0.001). Furthermore, naive CD4 T cells demonstrated a significant negative correlation with memory resting CD4 T cells (*r* = −0.6, *p*=0.002). Memory resting CD4 T cells were significantly negatively correlated with memory resting NK cells (*r* = −0.64, *p* < 0.001) and activated sendritic cells (*r* = −0.6, *p*=0.002), respectively. Moreover, memory-activated CD4 T cells were significantly positively correlated with gamma delta T cells (*r* = 0.82, *p* < 0.001) and macrophages M0 cells (*r* = 0.65, *p* < 0.001). The analysis also revealed a significant positive correlation between macrophages M0 cells and gamma delta T cells (*r* = 0.82, *p* < 0.001). In addition, resting NK cells exhibited a significant negative correlation with activated NK cells (*r* = −0.4, *p*=0.049). Moreover, macrophages M0 cells displayed a significant negative correlation with activated mast cells (*r* = −0.42, *p*=0.038). Furthermore, neutrophil cells were found to be significantly negatively correlated with activated mast cells (*r* = −0.46, *p*=0.025). Notably, rest mast cells were significantly positively correlated with neutrophil cells (*r* = 0.57, *p*=0.003) but significantly negatively correlated with activated mast cells (*r* = −0.41, *p*=0.046).

### 3.6. The Correlation Analysis Between B2M and C3 and Immune Cells

**Figure 7:**
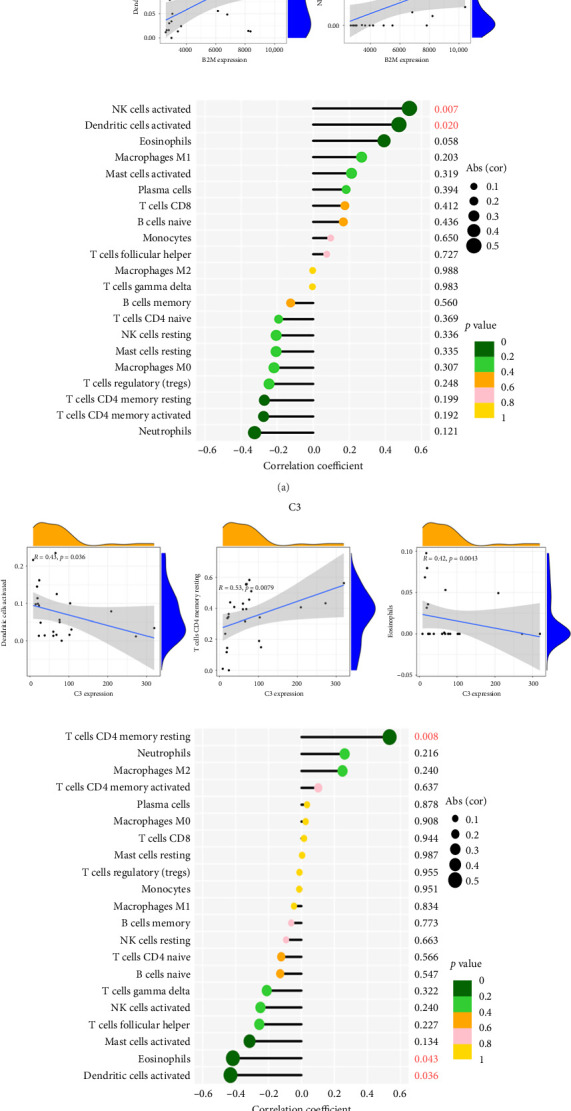
Correlation between B2M (a), C3 (b), and infiltrating immune cells in COVID-19 myocarditis.

The positive correlation analysis revealed that B2M, as depicted in Figure 7(a), exhibited a statistically significant positive correlation with activated NK cells (*r* = 0.53, *p*=0.0072) and activated dendritic cells (*r* = 0.48, *p*=0.02). Similarly, C3 exhibited a positive correlation with CD4 memory resting T cells (*r* = 0.53, *p* < 0.0079), while displaying a negative correlation with activated dendritic cells (*r* = −0.43, *p* < 0.036) and eosinophil cells (*r* = −0.42, *p*=0.043), as illustrated in Figure 7(b). These findings provide support for the conclusion that both B2M and C3 are associated with immune cell activity.

## 4. Discussion

Myocarditis, a disease that affects individuals of all ages groups, remains a prominent cause of fatalities associated with heart failure. Addressing this public health concern necessitates more rigorous investigation into its diagnosis, treatment, and recurrence screening [30]. Bioinformatics analysis has demonstrated its potential as a powerful tool for identifying novel targets that could serve as diagnostic and prognostic biomarkers. For instance, hub genes such as VSNL1, GABRA4, GABRB1, ACTG2, SCN1A, NFASC, AQP4, MYH6, COL1A1, and CSRP2 have been identified primarily clustering in extracellular matrix remodeling and sarcomere dysfunction in severe viral myocarditis cases. These genes are considered genetic markers of early high-risk myocarditis. By employing this computational discovery approach, a potential viable drug for preventing the progression of viral myocarditis to dilated cardiomyopathy has been identified as amlodipine [31]. Furthermore, a weighted gene co-expression network analysis (WGCNA) based on gene expression profiles from mouse models at different stages of viral myocarditis revealed the existence of three gene co-expression modules with the strongest correlation to acute or chronic disease stages. These modules were predominantly enriched in antiviral response and immune-inflammatory activation. The underlying hub genes and microRNAs derived from this analysis offer diagnostic value for both acute and chronic disease stages, and may aid in the development of new therapeutic targets for viral myocarditis [32]. Our study involved the collection of cohorts from GEO datasets based on two distinct virus-induced mouse models for myocarditis. Subsequent integrated analysis of data identified a total of 30 DEGs, including 16 upregulated genes and 14 downregulated genes. Functional enrichment analysis revealed the significant involvement of these DEGs in regulating immune effector processes, lymphocyte proliferation, MHC class II protein complex binding, antigen processing and presentation, B cell receptor signaling pathways, as well as coronavirus disease. Furthermore, the GSEA results demonstrated that the enriched pathways were predominantly associated with inflammatory response and interferon response. These findings are consistent with previous evidence implicating an inflammatory response in myocarditis pathogenesis.

The occurrence of myocardial damage resembling myocarditis was observed as one of the initial complications reported in COVID-19 patients during the early stages of the pandemic. It became evident early on that SARS-CoV-2 infection could lead to adverse cardiac events, including myocarditis [6, 33]. Because of heightened concerns regarding staff safety during the COVID-19 pandemic, EMB, which is typically required for diagnosing myocarditis in patients, was often not performed [34]. There is a significant imperative to investigate reliable and objective biomarkers for diagnosing COVID-19 myocarditis and comprehending its pathogenesis. This exploration is crucial for establishing a theoretical foundation to guide future clinical therapies. Despite the potential offered by high-throughput sequencing in identifying diagnostic markers for myocarditis, there remains a lack of bioinformatics-based analyses focusing on critical genes and mechanisms associated with COVID-19 myocarditis. The GSE167028 and GSE150392 datasets were subjected to a recent bioinformatic analysis, which unveiled six critical genes (CDK1, KIF20A, PBK, KIF2C, CDC20, and UBE2C) associated with COVID-19 myocarditis. This study also explored the underlying biological processes linking COVID-19 and myocarditis and demonstrated that SARS-CoV-2 contributes to myocarditis through pathophysiological mechanisms such as cell cycle regulation and ubiquitin-protein hydrolysis. Moreover, based on its findings, this study identified pertinent drugs for the clinical management of COVID-19 myocarditis [35]. In our current study, we employed a dataset comprising samples from COVID-19-associated cardiac pericytes to validate the expression levels of four selected features resulting from further analysis of DEGs using two distinct algorithms. Although there is limited abundance in cardiac cells, pericytes play a pivotal role in the process of infection. Among all cardiac cells, pericytes exhibit the highest expression of human angiotensin-converting enzyme 2 (ACE2), which serves as the primary entry point for the SARS-CoV-2 [36, 37]. Moreover, human cardiac pericytes are highly susceptible and permissive to SARS-CoV-2 infection facilitated by endosomal proteases, resulting in upregulation of inflammatory markers, vasoactive mediators, and nuclear factor kappa-B-dependent cell death [20]. Subsequently, significant dysregulation was observed in SARS-CoV-2 virus inoculated samples for two of these features: B2M and C3. These findings underscore the necessity for more comprehensive studies to enhance the effectiveness of biomarker screening for COVID-19 myocarditis. Furthermore, we assessed the diagnostic value of B2M and C3 in distinguishing COVID-19 myocarditis from control samples and found them to possess robust diagnostic capabilities, with an AUC of 0.978 (95% CI 0.911–1.000) for B2M and an AUC of 0.956 (95% CI 0.859–1.000) for C3. These results indicate the potential utility of these two biomarkers in effectively diagnosing COVID-19 myocarditis.

Beta-2-microglobulin (B2M) is a critical constituent of the major histocompatibility complex class I (MHC-I) molecule, expressed on the surface of nearly all nucleated cells [38]. This molecule plays a pivotal role in facilitating cell surface expression and maintaining structural stability of MHC-I, which is essential for antigen presentation and processing, regulation of inflammation, activation of the complement cascade, and response to stress [39, 40]. Importantly, epidemiological studies conducted in CVDs have consistently demonstrated a positive correlation between elevated B2M levels and an increased risk of CVD in both general population studies and individuals with renal conditions [41, 42]. A comprehensive meta-analysis incorporating 16 studies involving 30,988 participants and 5391 CVD events has confirmed moderate positive associations between B2M levels and the occurrence of CVD events as well as mortality [38]. Complement component 3 (C3) is the most abundant protein in the complement system and consists of an α- and a β-chain. Upon pathogen invasion or tissue damage, extracellular C3 can be spontaneously activated or cleaved by either C4b2a or C3bBb, resulting in the formation of C3a and C3b. The former can activate the C3a receptor (C3aR) on cells, thereby inducing either pro- or anti-inflammatory responses [43–45]. In relation to CVDs, a study conducted on mice lacking C3 concluded that it may protect the heart against ischemia-reperfusion injury through its involvement in critical metabolic pathways for energy production and cell survival [43]. Furthermore, a recent study demonstrated that genetic depletion of both C3 and C5 significantly prevents spontaneous myocarditis, while using small-interfering RNA targeting C3 effectively prevents onset of myocarditis and improves the recovery of heart functions in experimental autoimmune myocarditis (EAM) [46]. Interestingly, a contrasting finding emerged from another study indicating that an unfavorable prognosis in COVID-19 patients is associated with excessive activation of the complement system, which seemingly contradicts our findings [47]. We postulate that this discrepancy may stem from variances in the sources of sequencing data samples. Another plausible explanation could be the potential correlation between C3 expression levels and viral load, as well as variations in C3 expression across different stages of the disease.

Myocarditis is a complex inflammatory disease affecting the heart, involving a diverse range of immune cell types in the immune response. Therefore, it is crucial to assess immune infiltration and determine whether differences in the composition of immune infiltration can contribute to the development of novel immunotherapeutic strategies. The CIBERSORT algorithm serves as a valuable analytical tool for evaluating changes in immune cell expression using RNA-seq data, enabling identification of the proportions of various immune cell types present in samples [48]. Analysis utilizing the CIBERSORT algorithm on five pediatric patients with myocarditis revealed that activated NK cells were predominant in two patients, while activated memory CD4+ T cells and M2 macrophages each predominated in one patient. These findings were consistent with those obtained from immunohistochemistry, potentially reflecting the temporal dynamics of innate and adaptive immune responses during acute myocarditis [49]. Dermatomyositis (DM) is an acquired autoimmune disease that can lead to myocardial damage, although the underlying mechanisms remain incompletely understood. An integrated bioinformatics analysis of CIBERSORT explored immune cell infiltration in myocardium specimens of myocarditis. This finding revealed an increased presence of M2 macrophages compared to healthy controls, suggesting a potential crucial role for these feature genes (IFIT3, OAS3, ISG15, and RSAD2) in the underlying mechanism of myocardial injury in DM [50]. However, limited research has utilized CIBERSORT-based analyses on immune infiltration related to COVID-19-associated myocarditis. In our present study, we employed the CIBERSORT algorithm and predicted significant associations between proportions of activated NK cells, macrophage M1 cells, activated dendritic cells, and neutrophil cells within SARS-CoV-2 infected samples. Furthermore, B2M exhibited a positive correlation with activated NK cells and activated dendritic cells while C3 displayed positive correlations with CD4 memory resting T cells but negative correlations with activated dendritic cells and eosinophil cells. These findings have the potential to provide valuable insights into comprehensive characterization of infiltrating immune cell populations in myocarditis as well as offer novel perspectives on the pathogenesis of COVID-19-associated myocarditis.

Furthermore, this study highlights areas that require further investigation. One limitation to consider is the small sample size in the cohort. In addition, it is important to acknowledge the inherent limitations of CIBERSORT, as it tends to systematically overestimate or underestimate certain cell types despite exhibiting a lower estimation bias compared to other methods [51]. Moreover, the absence of protein expression data in our study is worth noting. Furthermore, it is imperative to reinforce the conclusions derived from RNA-seq or microarray analysis with RT-PCR within larger clinical samples in order to enhance the validity of our findings.

## 5. Conclusions

In summary, diagnostic biomarkers for COVID-19 myocarditis encompass B2M and C3. The pathogenesis of COVID-19 myocarditis potentially involves neutrophils, activated dendritic cells, M1 macrophages, and activated NK cells. These immune cell populations represent prospective targets for immunotherapy in patients with COVID-19 myocarditis.

## Data Availability

Publicly available datasets were analyzed in this study. These data can be found here: GSE53607 (https://www.ncbi.nlm.nih.gov/geo/query/acc.cgi?acc=GSE53607), GSE35182 (https://www.ncbi.nlm.nih.gov/geo/query/acc.cgi?acc=GSE35182), and GSE183850 (https://www.ncbi.nlm.nih.gov/geo/query/acc.cgi?acc=GSE183850). The inquiries of original contributions presented in the study can be directed to the corresponding authors.
